# Magnonic band structure investigation of one-dimensional bi-component magnonic crystal waveguides

**DOI:** 10.1186/1556-276X-7-498

**Published:** 2012-09-04

**Authors:** Fu Sheng Ma, Hock Siah Lim, Vanessa Li Zhang, Ser Choon Ng, Meng Hau Kuok

**Affiliations:** 1 Department of Physics, National University of Singapore, Singapore, 117542, Singapore

**Keywords:** Magnonic crystal, Magnonics, Spin wave, Bandgap, Micromagnetic simulations, 75.40.Gb, 75.30.Ds, 75.78.Cd, 75.78.-n

## Abstract

The magnonic band structures for exchange spin waves propagating in one-dimensional magnonic crystal waveguides of different material combinations are investigated using micromagnetic simulations. The waveguides are periodic arrays of alternating nanostripes of different ferromagnetic materials. Our results show that the widths and center frequencies of the bandgaps are controllable by the component materials, the stripe widths, and the orientation of the applied magnetic field. One salient feature of the bandgap frequency plot against stripe width is that there are *n*-1 zero-width gaps for the *n*th bandgap for both transversely and longitudinally magnetized waveguides. Additionally, the largest bandgap widths are primarily dependent on the exchange constant contrast between the component materials of the nanostructured waveguides.

## Background

Bandgap and dispersion relation are two important properties of photonic crystals (PhCs) to control light and electromagnetic waves. PhCs are believed to play a key role of future core components of novel electro optical applications. The potential applications extend from simple waveguides or splitters over multiple wavelength demultiplexers and wavelength filters to advanced applications such as single photon sources or laser resonators. Future sensor and data processing industry may profit out of PhCs as well as the telecommunication sector. PhCs with dimensions in the low sub-100-nm region [1,2], and even in the IR range [3], have already been successfully fabricated using high-resolution electron beam lithography and nanoimprint lithography. Magnonic crystals (MCs) [4-12], as the magnetic counterpart of PhCs, also exhibit the bandgap and dispersion relation properties that can be exploited to control and manipulate spin waves (SWs) and also to produce effects that are not possible with isolated magnetic nanostructures. The bandgap property can forbid propagation of a certain frequency range of SWs into MCs. Since wavelengths of SWs span several orders of magnitude from tens of microns to below 1 nm, their frequencies may vary from gigahertz to terahertz. Additionally, the frequency for a given wavelength can be shifted by the magnetic field. This broad region in length and time scales is one reason that makes SWs so interesting for high-frequency applications.

The SWs in MCs can be classified according to the magnitude of their wavelength as dipolar- or exchange-dominated SWs [9]. This is essentially because dipole-dipole interaction is long-ranged, while exchange interaction is short-ranged. For wavelengths longer than 1 μm [13], the dispersion of the SWs is dominated by dipolar interactions. For the in-plane magnetized thin-film magnetic nanostructures, the dipolar-dominated SWs can be classified into magnetostatic surface spin wave (MSSW) and backward volume magnetostatic spin wave (BVMSW) modes depending on their propagation direction with respect to the magnetization [14]. The MSSW modes, whose frequencies lie above the spatially uniform precession (Kittel) mode, are characterized by perpendicular wave propagation relative to the applied in-plane magnetic field. In contrast, the BVMSW modes, with a precession frequency smaller than that of the Kittel mode, are characterized by wave propagation parallel to the applied field. Since BVMSW modes travel ‘backward’ in phase, this leads to a negative dispersion. Therefore, if dipolar interactions dominate, the band structure will be anisotropic with regard to the applied field direction: the positive dispersion for MSSW mode and the negative dispersion for BVMSW mode. For wavelengths below 1 μm [13], the exchange interaction becomes important so that this contribution has to be taken into account for mixed dipole and exchange spin waves in an intermediate region of length scales. For wavelengths below 100 nm [13], the magnetostatic contribution to the energy of SWs will be dominated by the exchange interaction. Hence, the dispersion is dominated by the exchange interaction. As a consequence of neglecting the anisotropic dipolar contribution, the dispersion in the exchange limit is positive for the two relative orientations between wave propagation and magnetization direction.

Recently, the dispersion relations of SWs in one-dimensional (1D) MCs, with lattice constants in the order of several hundreds of nanometers or several micrometers, have been investigated. These MCs consist of dipolarly coupled nanostripes [15-17] and of contacting alternating stripes of two different materials [10,11,18,19], and the magnonic band structures of SWs are dominated by dipolar interactions. Among these MCs of lattice constant larger than 200 nm, the largest widths of transmission band and forbidden band (or bandgap) are 2.5 and 2.1 GHz, respectively. Larger values are expected for MCs of lattice constant smaller than 100 nm, in which the magnonic band structures of SWs are dominated by exchange interactions. However, scarce attention has been paid to the magnonic band structures of exchange SWs in MCs with lattice constant in the order of tens of nanometers [9]. Although the fabrication of MCs with nanoscale lattice constants and the detection of exchange SWs are still challenging, the high frequency and short wavelength gave exchange SW an advantage over the dipolar-dominated SW. In this work, we present the results of a micromagnetic study of magnonic band structures for exchange SWs propagating in 1D bi-component magnonic crystal waveguides (MCWs). The waveguides are periodic arrays of alternating stripes of different ferromagnetic materials. The properties of the bi-component bandgaps are studied as a function of the constituent components, the stripe width to lattice constant ratio, and also the applied field orientation.

## Methods

**Figure 1:**
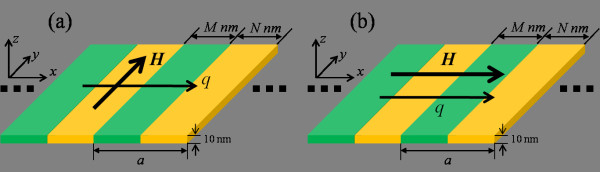
** Schematic of the magnonic crystal waveguide comprising of alternating nanostripes of two ferromagnetic materials.** The lattice constant *a* = *M* + *N*, where *M* and *N* are the respective widths of the stripes of the two materials. A magnetic field *H* is applied (**a**) transversely and (**b**) longitudinally to the waveguide, and ***q*** is the wave vector of the SWs.

We consider a MCW with a length 1,000 nm (*x* direction) in the form of laterally patterned periodic arrays of alternating stripes of different ferromagnetic materials (Figure 1). Each stripe in the MCW has a length of 140 nm (*y* direction) and a thickness of 10 nm (*z* direction). We investigated the magnetization dynamics of bi-component MCWs each composed of two of the following ferromagnetic metals, namely Co, Fe, Permalloy (Py), and Ni, in a total of six possible combinations. For brevity, the MCWs with respective stripe widths of *M* and *N* nm of different ferromagnetic materials are referred to as *M*Co/*N*Ni, *M*Co/*N*Py, *M*Co/*N*Fe, *M*Fe/*N*Ni, *M*Fe/*N*Py, and *M*Py/*N*Ni. Two kinds of SW modes, which depend on the orientation of the applied magnetic field *H*, in transversely and longitudinally magnetized waveguides were investigated as shown in Figure 1a,b, respectively. The magnonic band structures of exchange SWs with wavelengths down to several nanometers and frequencies up to hundreds of gigahertz are numerically investigated.

The Object Oriented Micromagnetic Framework program [19] was used to numerically calculate the dynamics of the magnetizations by solving the Landau-Lifshitz-Gilbert equation [20,21]. The simulation cell size used is 2 × 2 × 10 nm^3^, the damping constant *α* = 0.01, and the gyromagnetic ratio *γ* = 2.21 × 10^5^ m/As. The magnetic parameters of the four ferromagnetic metals (Co, Fe, Py, and Ni) used in the simulations are specified in Table 1. A static in-plane magnetic field was applied along the *y* and *x* directions (see Figure 1) corresponding to the transverse and longitudinal geometries, respectively. In order to excite SWs, a ‘sinc’ function [22] with *H*_0_ = 1.0 T and field frequency *f*_*H*_ = 250 GHz was applied locally to a volume element Δ*x*Δ*y*Δ*z* (=10 × 140 × 10 nm^3^) in the middle of the MCWs (*x* = 500 nm). SWs, with frequencies ranging from 0 to 250 GHz, were thus excited and propagated along the *x* direction. The dispersion curves of SWs propagating along the length (*x* direction) of the MCWs were calculated using the following procedures [12]. First, the time dependence of the magnetization of each cell is recorded. The dispersion curve for the propagation of spin waves is then obtained by performing a 2D Fourier transform of the out-of-plane component of the magnetization in space and time.

**Table 1:** Magnetic parameters of ferromagnetic metals Co, Fe, Py, and Ni

**Metals**	***M***_***s***_**(10**^**6**^ **A/m)**	***A*(10**^**−11**^ **J/m)**	***l***_**ex**_**(nm)**
Co	1.445	3.00	4.78
Fe	1.752	2.10	3.30
Py	0.860	1.30	7.64
Ni	0.484	0.86	5.29

*M*_s_, saturation magnetization; *A*, exchange constant; *l*_ex_, exchange length [6].

## Results and discussion

### Transversely magnetized MCWs

**Figure 2:**
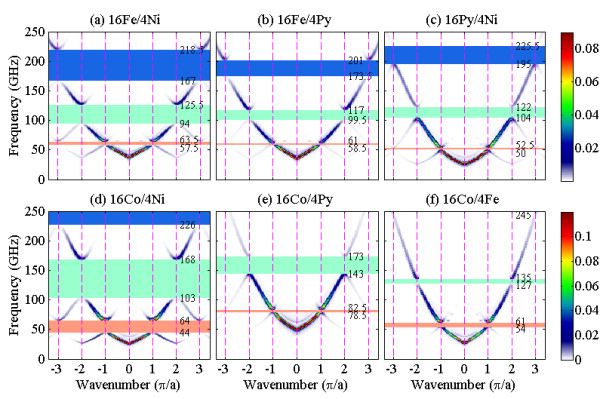
** Dispersion relations for transversely magnetized MCWs.** (**a**) 16Fe/4Ni, (**b**) 16Fe/4Py, (**c**) 16Py/4Ni, (**d**) 16Co/4Ni, (**e**) 16Co/4Py, and (**f**) 16Co/4Fe MCWs under a *H* = 600 mT field applied along the *y* axis. The dotted lines indicate the Brillouin zone boundaries ***q*** = *n*π/*a*, and the first, second, and third bandgaps are denoted by the red-, green-, and blue-shaded regions, respectively. The intensities of the SWs are represented by color scale.

For the transversely magnetized waveguides (see Figure 1a), the calculated SW dispersion curves along the longitudinal symmetry axis of 16Fe/4Ni, 16Fe/4Py, 16Py/4Ni 16Co/4Ni, 16Co/4Py, and 16Co/4Fe MCWs for *H* = 600 mT are shown in Figure 2. Due to the effect of width confinement [22-24], the dispersion curves are characterized by a lower frequency limit, *viz.* 32.0 GHz for 16Fe/4Ni, 31.5 GHz for 16Fe/4Py, 21.0 GHz for 16Py/4Ni, 25.0 GHz for 16Co/4Ni, 43.0 GHz for 16Co/4Py, and 21.5 GHz for 16Co/4Fe MCWs. These values correspond to the minimum frequency of the lowest allowable SW modes propagating through the respective waveguides. In contrast to the single monotonic dispersion curve of the isolated nanostripes [22], the periodic character of the three dispersion branches of the MCWs, calculated up to the third Brillouin zone (BZ), is evident from Figure 2. The dispersion curves are observed to be folded and exhibit bandgaps at the BZ boundaries (*q* = *n*π/*a*, *n* = 1, 2, and 3) due to the periodic modulation of the material magnetic properties along the SW propagation direction. For the 16Fe/4Ni MCW, the first three bandgaps with respective widths of 6.0, 31.5, and 51.5 GHz exhibited at the BZ boundaries are shown in Figure 2a. Another notable feature is the variation of the SW mode intensities of the three branches over the three BZs, which are proportional to the squared Fourier transform of the dynamic magnetization [25]. The lowest branch has the maximum intensity in the first BZ, the second one in the second BZ, and so on. This is a consequence of the Umklapp process, which involves the reciprocal lattice vector *G* (*G* = *n*2π/*a*) [11]. By comparing the widths and center frequencies of the bandgaps for the six MCWs studied as shown in Figure 2a,b,c,d,e,f, it is interesting to note that the width and center frequencies of bandgaps are dependent on the component materials of the MCWs.

**Figure 3:**
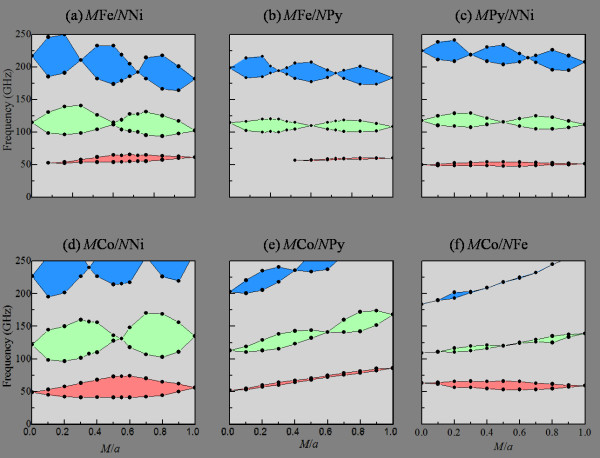
** Bandgap diagram with respect to*M/a*under applied field*H*** **= 600 mT.** With *a* (=*M* + *N*) = 20 nm for transversely magnetized (**a**) *M*Fe/*N*Ni, (**b**) *M*Fe/*N*Py, (**c**) *M*Py/*N*Ni, (**d**) *M*Co/*N*Ni, (**e**) *M*Co/*N*Py, and *M*Co/*N*Fe MCWs. The gray region represents the allowed bands, while the red, green, and blue regions, the respective first, second, and third forbidden bands.

Simulations were also carried out for transversely magnetized MCWs to study the dependence of the bandgaps on the width *M* of a stripe that has a larger exchange constant in the MCW with the lattice constant *a* (= *M* + *N*) kept fixed at 20 nm. The characteristics of the first three bandgaps, obtained at the BZ boundaries *q* = π/*a*, 2π/*a*, and 3π/*a*, as a function of *M/a* for *H* = 600 mT for the six MCWs are displayed in Figure 3. In Figure 3a, the first bandgap of *M*Fe/*N*Ni MCWs exists over almost the entire range of Fe stripe widths from 0 to 20 nm, and its maximum width of 11 GHz occurs at *M*/*a* = 0.6. The second bandgap has a zero-gap (i.e., bandgap with a zero-width) at *M*/*a* = 0.5 and two local maximal widths, *viz*. 43.0 GHz at *M*/*a* = 0.2 and 36.0 GHz at *M*/*a* = 0.7. For the third bandgap, there are two zero-gaps at *M*/*a* = 0.3 and 0.65 and three local maximal widths, *viz*. 61.5 GHz at *M*/*a* = 0.1, 59 GHz at *M*/*a* = 0.5, and 52 GHz at *M*/*a* = 0.8. This implies that there are *n*-1 zero-gaps for the *n*th bandgap. This feature is also exhibited by all the other MCWs as shown in Figure 3a,d,c,d,e,f.

### Longitudinally magnetized MCWs

**Figure 4:**
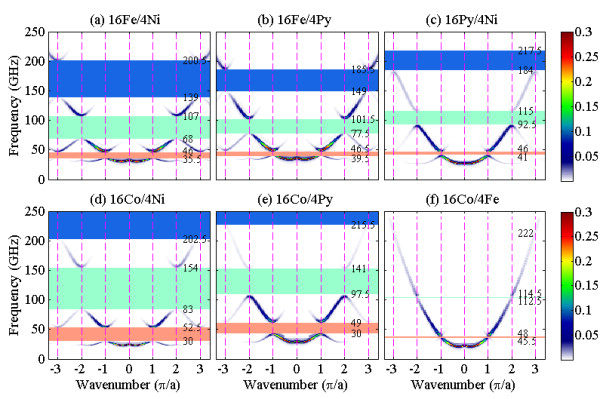
** Dispersion relations for longitudinally magnetized MCWs.** (**a**) 16Fe/4Ni, (**b**) 16Fe/4Py, (**c**) 16Py/4Ni, (**d**) 16Co/4Ni, (**e**) 16Co/4Py, and (**f**) 16Co/4Fe MCWs under a *H* = 600 mT field. The dotted lines indicate the Brillouin zone boundaries ***q*** = *n*π/*a*, and the first, second, and third bandgaps are denoted by the red-, green-, and blue-shaded regions, respectively. The intensities of the SWs are represented by color scale.

For the longitudinally magnetized waveguides (see Figure 1b), the calculated SW dispersion curves along the longitudinal symmetry axis of 16Fe/4Ni, 16Fe/4Py, 16Py/4Ni 16Co/4Ni, 16Co/4Py, and 16Co/4Fe MCWs for *H* = 600 mT are shown in Figure 4. Due to the effect of width confinement [22-24], the dispersion curves are characterized by a lower frequency limit, *viz.* 25.0 GHz for 16Fe/4Ni, 28.5 GHz for 16Fe/4Py, 21.5 GHz for 16Py/4Ni, 21.5 GHz for 16Co/4Ni, 25.0 GHz for 16Co/4Py, and 27.0 GHz for 16Co/4Fe MCWs. These values correspond to the minimum frequency of the lowest allowable SW modes propagating through the respective waveguides. Similar to the transverse case, the characteristics of bandgaps are also dependent on the component materials of the MCWs. For the 16Fe/4Ni MCW, the first three bandgaps with respective widths of 10.5, 39.0, and 61.5 GHz are shown in Figure 4a.

Unlike the dispersion curves of transversely magnetized waveguides, a negative dispersion is observed near the BZ center (*q* = 0) where SWs with small wave vectors are dominated by long-range dipolar interaction. The dispersion curves of dipole-dominated SWs are positive or negative depending on whether the orientation of wave propagation is transverse or parallel to the external field. In contrast, the dispersion curves of SWs with a large wave vector whose properties are dominated by short-range exchange interaction are positive and independent of the orientation of the external field. Therefore, the SW modes near the first BZ boundary are exchange spin waves. It is interesting to note that the widths of the first three bandgaps in the longitudinally magnetized 16Co/4Fe MCW (see Figure 4f) are narrower than the corresponding ones of the transversely magnetized case (*cf*. Figure 2f). However, the bandgaps of the other five MCWs (see Figure 4a,b,c,d,e) are wider than those of the corresponding transversely magnetized ones (see Figure 2a,b,c,d,e). For instance, the width of the first bandgap for longitudinally (transversely) magnetized 16Co/4Fe MCW is 2.5 GHz (7.0 GHz), while the width of the first bandgap for longitudinally (transversely) magnetized 16Fe/4Ni MCW is 10.5 GHz (6.0 GHz).

**Figure 5:**
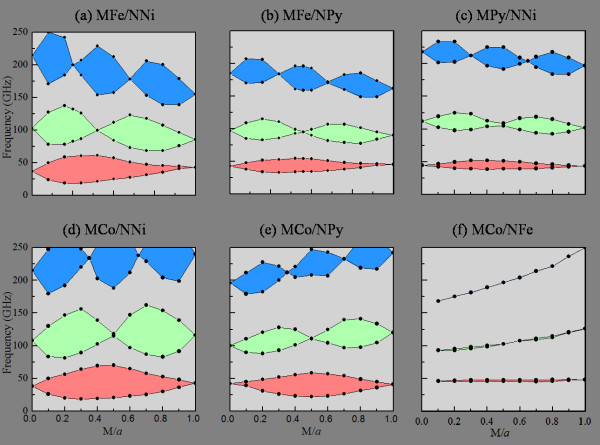
** Bandgap diagram with respect to*M/a*for longitudinally magnetized MCWs.** With *a* (=*M* + *N*) = 20 nm for (**a**) *M*Fe/*N*Ni, (**b**) *M*Fe/*N*Py, (**c**) *M*Py/*N*Ni, (**d**) *M*Co/*N*Ni, (**e**) *M*Co/*N*Py, and (**f**) *M*Co/*N*Fe MCWs under applied field *H* = 600 mT. The gray regions represent the allowed bands, while the red, green, and blue regions, the respective first, second, and third forbidden bands.

The dependencies of the magnonic bandgaps on the width *M* of the stripe with the lattice constant *a* (=*M* + *N*) kept fixed at 20 nm were also investigated for longitudinally magnetized MCWs. The characteristics of the first three bandgaps, obtained at the BZ boundaries (*q* = π/*a*, 2π/*a*, and 3π/*a*) as a function of *M/a* for *H* = 600 mT, are displayed in Figure 5. The feature reported for transversely magnetized MCWs, *viz.* that the *n*th bandgap has *n*-1 zero-gaps, is also exhibited by all the six MCWs, as shown in Figure 5. For the *M*Fe/*N*Ni MCWs, the first bandgap exists over almost the entire range of Fe stripe widths from 0 to 20 nm, and its maximal width of 42.0 GHz appears at *M*/*a* = 0.3. For the second bandgap, there is one zero-gap at *M*/*a* = 0.4 and two local maximal widths, *viz*. 59.0 GHz at *M*/*a* = 0.2 and 49.5 GHz at *M*/*a* = 0.7. For the third bandgap, there are two zero-gaps at *M*/*a* = 0.25 and 0.6 and three local maximal widths, *viz*. 79.0 GHz at *M*/*a* = 0.1, 75.0 GHz at *M*/*a* = 0.4, and 61.5 GHz at *M*/*a* = 0.8.

**Figure 6:**
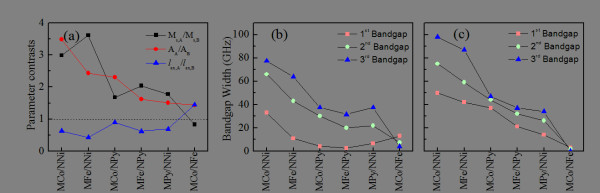
** Calculated maximal widths of the first three bandgaps.** (**a**) Magnetic parameter contrasts between component materials in the MCWs, which are arranged in order of decreasing exchange constant ratio. The dashed line represents the contrast ratio equal to 1. Maximal widths of the magnonic bandgap for (**b**) transversely and (**c**) longitudinally magnetized MCWs of all the considered material combinations under applied field *H* = 600 mT.

The calculated maximal widths of the first three bandgaps are shown in Figure 6. In Figure 6a, magnetic parameter contrasts (static magnetization contrast, *M*_s,A_/*M*_s,B_, exchange constant contrast, *A*_A_/*A*_B_, and exchange length contrast, *l*_ex,A_/*l*_ex,B_) between the component materials of the MCWs are plotted. The calculated maximum widths of the first three bandgaps of the transversely and longitudinally magnetized MCWs are shown in Figure 6b,c, respectively. As shown in Figure 6b,c, the maximum widths of the bandgaps are dependent on the component materials of the MCWs.

It can be seen from Figure 6b that the largest first, second, and third bandgaps among the MCWs studied are all observed in the *M*Co/*N*Ni MCWs, which have the largest exchange constant ratio. The smallest second and third bandgaps are observed in the *M*Co/*N*Fe MCWs (7.5 and 4.0 GHz), which has the smallest exchange constant ratio. Interestingly, however, the smallest first bandgap is observed in the *M*Fe/*N*Py MCWs (2.5 GHz).

For the longitudinally magnetized MCWs (Figure 6c), the maximum widths of the first three bandgaps monotonically decrease with decreasing exchange constant ratio. Unlike the transversely magnetized waveguides, the smallest first, second, and third bandgaps are all observed in the *M*Co/*N*Fe MCWs (3.0, 2.0, and 0.0 GHz). In general, the larger the exchange constant contrast between the material components of a nanoscale bi-component MCW, the wider would be its bandgap.

A closer inspection of Figure 6b,c reveals two unusual behaviors in *M*Co/*N*Fe MCW in comparison with the other five MCWs. Firstly, its higher-order bandgaps has a narrower width than those of the lower-order bandgaps, in sharp contrast with the other five MCWs. Secondly, the bandgaps of transversely magnetized *M*Co/*N*Fe MCWs are wider than the corresponding ones in the longitudinally magnetized case, and the other five MCWs, on the other hand, exhibit a completely opposite behavior.

The contrasting behaviors between the *M*Co/*N*Fe MCWs and the other five types of MCWs may be attributed to the magnetic parameter contrasts between the component materials of the MCWs. From Figure 6a, it can be seen that the *M*Co/*N*Fe MCWs have an exchange length contrast larger than 1, but its magnetization contrast is smaller than 1. The other five types of MCWs, on the other hand, have static magnetization contrast larger than 1, but the exchange length contrast smaller than one.

## Conclusions

The magnonic band structures of exchange SWs in 1D bi-component magnonic crystal waveguides were investigated using the micromagnetic methods. Two kinds of SWs were studied according to the relative orientation between the applied field and the waveguides: the transverse and the longitudinal cases. From the calculated dispersion curves of SWs, we found that the widths and center frequencies of the bandgaps are controllable by the component materials, the stripe width to lattice constant ratio as well as the orientation between the applied field and the waveguide. A striking feature of the dispersion curve is that there are *n*-1 zero-gaps for the *n*th bandgap for both the transverse and longitudinal cases. The largest bandgap widths were observed in the *M*Co/*N*Ni MCWs, which have the largest exchange constant ratio. By comparing the band structures of exchange SWs in both the transverse and the longitudinal cases, we found that for the same MCW, the widths of the bandgaps in the longitudinal case are wider than those in the transverse case except for the *M*Co/*N*Fe MCWs. The investigation of MCs with nanoscale periods, in which SW frequencies can reach values up to the terahertz range and with wavelengths of just a few nanometers, opens the way to practical applications of the dynamic properties of such MCs in much faster devices of nanometer size.

## Competing interests

The authors declare that they have no competing interests.

## Authors' contributions

FSM carried out the calculations and analyses. HSL designed the project and participated in the analyses. VLZ, SCN, and MHK helped in the interpretation of the results and drafting of the manuscript. All authors read and approved the final manuscript.
